# A Case

**Published:** 1865-12

**Authors:** 


### A CASE.

---

In the latter part of May, 1865, a patient presented himself for treatment, having a soft tumor the size of a quail's egg, in the roof of his mouth. His teeth appeared to be sound. A central and lateral incisor had been extracted, though not decayed, as he told me, some months before I saw him. On opening the tumor, a free discharge of sero-purulent matter followed, the odor of which was exceedingly offensive. On examination, a probe passed readily into the antrum, and through the floor of the nostril. I removed a portion of the internal alveolar process, which was necrosed, and partly detached.

The cavity of the tumor, and the antrum, were freely washed with a mixture of equal parts of liquor sodæ chlorinatæ and water; and a pledget of lint, saturated with the same, was left in the orifice of the tumor. The dressings, for a while, were changed daily, but less and less frequently as the discharge of matter abated. After a lapse of ten or twelve days, I used, once a week, an injection of tincture of iodine. This will give a general idea of the local treatment.

The patient had suffered from chancre, followed by secondary symptoms, about three years before. I regarded the condition of his mouth as an indication of tertiary syphilis, and, consequently put him on a course of iodide of potassium. The case progressed satisfactorily, till the latter part of July, when I found the teeth had become much more firm in their sockets, and the tumor was discharging healthy pus. On account of my own illness, I saw no more of him till near the last of October, when he called on me, apparently in excellent health, the tumor gone, the orifice closed, the teeth firm, and the bony tissue apparently replaced by new formation, of the proper form and dimensions. He had taken the iodide every day, from the time it was first prescribed.

When I removed the necrosed bone, a small scratch on my left hand was inoculated with the matter that surrounded it. As the scratch had not been observed, the accident was not noticed at the time. By the next morning, the hand and arm were swollen and painful. Erysipelatous manifestations, from the arm to the elbow, were present. The mouth was dry and feverish, the pulse about a hundred, with headache and general constitutional disturbance. These symptoms continued, and increased for over a week, after which there was a gradual return to health.

In all such cases, abrasions of the skin should be covered with tissue paper, or lint, and collodion. Prevention is more profitable, as well as more pleasant than cure.

